# De Novo Design of Parallel and Antiparallel A_3_B_3_ Heterohexameric α-Helical Barrels

**DOI:** 10.1021/acs.biochem.4c00584

**Published:** 2025-04-14

**Authors:** Joel J. Chubb, Katherine I. Albanese, Alison Rodger, Derek N. Woolfson

**Affiliations:** † School of Chemistry, University of Bristol, Cantock’s Close, Bristol BS8 1TS, U.K.; ‡ School of Natural Sciences, Macquarie University, Sydney, New South Wales 2019, Australia; § Research School of Chemistry, Australian National University, Canberra, ACT 2601, Australia; ∥ Max Planck-Bristol Centre for Minimal Biology, University of Bristol, Cantock’s Close, Bristol BS8 1TS, U.K.; ⊥ School of Biochemistry, University of Bristol, Medical Sciences Building, University Walk, Bristol BS8 1TD, U.K.; # Bristol BioDesign Institute, University of Bristol, Cantock’s Close, Bristol BS8 1TS, U.K.

## Abstract

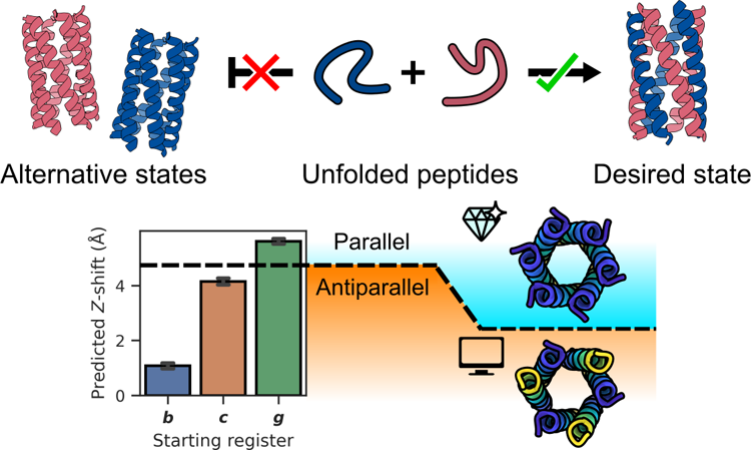

The de novo design of α-helical coiled-coil peptides is advanced. Using established sequence-to-structure relationships, it is possible to generate various coiled-coil assemblies with predictable numbers and orientations of helices. Here, we target new assemblies, namely, A_3_B_3_ heterohexamer α-helical barrels. These designs are based on pairs of sequences with three heptad repeats (***abcdefg***), programmed with ***a*** = Leu, ***d*** = Ile, ***e*** = Ala, and ***g*** = Ser, and ***b*** = ***c*** = Glu to make the acidic (A) chains and ***b*** = ***c*** = Lys in the basic (B) chains. These design rules ensure that the desired oligomeric state and stoichiometry are readily achieved. However, controlling the orientation of neighboring helices (parallel or antiparallel) is less straightforward. Surprisingly, we find that assembly and helix orientation are sensitive to the length of the overhang between helices. To study this, cyclically permutated peptide sequences with three heptad repeats (the register) in the peptide sequences were analyzed. Peptides starting at ***g*** (***g***-register) form a parallel 6-helix barrel in solution and in an X-ray crystal structure, whereas the ***b***- and ***c***-register peptides form an antiparallel complex. In lieu of experimental X-ray structures for ***b***- and ***c***-register peptides, AlphaFold-Multimer is used to predict atomistic models. However, considerably more sampling than the default value is required to match the models and the experimental data, as many confidently predicted and plausible models are generated with incorrect helix orientations. This work reveals the previously unknown influence of the heptad register on helical overhang and the orientation of α-helical coiled-coil peptides and provides insights for the modeling of oligopeptide coiled-coil complexes with AlphaFold.

## Introduction

The de novo design of proteins and protein-like peptide assemblies has advanced significantly in recent years.^1−3^ Over the past two decades, we and others have aimed to understand and parametrize a particular type of peptide assembly and protein fold called α-helical coiled coils (CCs).^4−6^ This has resulted in a guided exploration of CC sequence and structure space with de novo-designed peptide and protein assemblies.^7−11^ From this, we have developed a set of rules to design peptides that assemble into a wide range of CC structures.

CCs comprise two or more α helices that wrap around one another like threads of rope, creating a larger super helix.^12−14^ A key component of CC assembly is that the hydrophobic side chains of one α helix project into diamond-shaped holes formed between side chains of a neighboring helix. These are called knob-into-hole (KIH) interactions, and they specify CC structures in terms of oligomeric state, helix-partner preferences, and helix orientation.^4^ KIH interactions are encoded by sequence repeats of hydrophobic (***h***) and polar residues (***p***), (***hpphppp***), often called heptads and labeled ***abcdefg***. It is the ***h*** residues at ***a*** and ***d*** that form the main hydrophobic KIH interactions, but, as described below, residues at ***e*** and ***g*** also contribute to the helical interfaces through additional hydrophobic and salt-bridging interactions. Typically, stable CC peptide assemblies require three or more contiguous heptad repeats, and the canonical ***hpphppp*** repeats with charged residues at ***e*** and ***g*** tend to form dimers, trimers, or tetramers.

α-Helical barrels (αHBs) are a subset of CCs in which five or more α-helices assemble to generate central solvent-accessible channels along the superhelical axis.^8,15^ Most αHBs are programmed by variations of the heptad repeat with further interfacial, ***h*****-**like residues at the ***e*** and ***g*** sites to give ***hpphhph*** repeats, though in smaller αHBs, polar side chains like threonine and serine can be accommodated. Over the past decade, we have determined clear sequence-to-structure relationships that define the oligomeric states for specific assemblies of 5–9 helices, which can be used as rules for rational and computational de novo peptide and protein design.^4,8,9,11^ These rules are exemplified in the next section for hexameric barrels. The de novo αHBs presented to date are also highly thermostable, making them exciting prospects as scaffolds for functional protein design. Indeed, they have been used for small-molecule sensing, ion transport across membranes, and rudimentary catalysis.^16−18^ Recently, single-chain variants of αHBs with 5–8 helices with pseudocyclic symmetry have been generated by using the sequence-to-structure relationships as seeds for AI-based computational design.^11^

**Figure 1:**
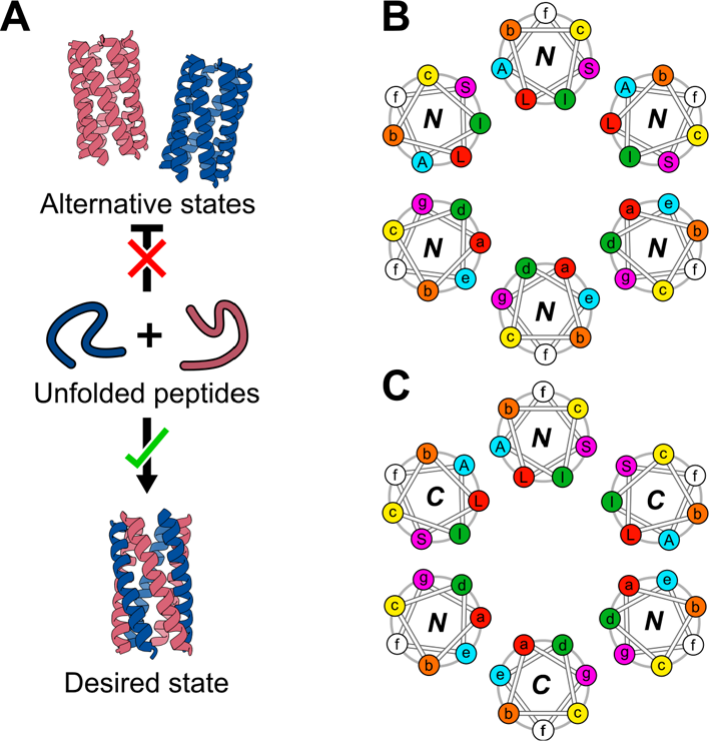
Design goal and features of coiled coils. (A) Our proposed design incorporates two differently charged peptides—an acidic peptide (red) and a basic peptide (blue)—that should not self-associate but fold only when both peptides are present into a 6-helix barrel. (B) Helical-wheel diagram for a 6-helix barrel with parallel helix orientations. Here, the ***a*** sites (and hence all heptad positions) are aligned with themselves on the *Z*-axis. The ***g-a-d-e*** positions in the top half of the helical wheels are embellished with the amino acids placed at those positions: *S-L-I-A.* (C) Helical-wheel diagram of a 6-helix barrel with antiparallel helix orientations. Here, the ***a*** sites are more closely aligned vertically to the ***d*** sites. The ***g-a-d-e*** positions in the top half of the helical wheels are embellished with the amino acids placed at those positions: *S-L-I-A*.

While heteromeric dimeric, trimeric, and tetrameric CCs have been designed,^10,19−24^ most of de novo-designed αHB assemblies are homomeric. For functionalization and applications, heteromeric assemblies have advantages including controlled assembly—i.e., conditional folding dependent on the presence of two or more different peptide/protein chains—and a reduction in sequence and structural symmetry, allowing subsets of the peptides to be modified. Mostly, heteromeric de novo CCs have been targeted by using different charged variants of the peptide chains to promote discriminating electrostatic interactions between the different chains. For instance, mixtures of acidic (A, anionic) and basic (B, cationic) peptides have been used to create AB-type dimers, ABC trimers, and A_2_B_2_ tetramers.^19−21,25−28^ Others have used cofactors, steric packing, and hydrogen-bonding networks to generate heteromeric CCs.^24,29−31^ However, these require mutation of core-packing residues. There have been some designs of A_3_B_3_ αHBs and bundles; however, our previous attempt to do this gave homomeric assemblies of either the A or B peptides as well as the desired heteromeric barrels, suggesting that the homomers and heteromers are of similar stability and therefore present as a highly heterogeneous mixture of the three potential species in equilibrium, which complicates the system for downstream applications (Figure 1).^32^ Similar behavior is observed for a heterohexameric bundle designed by Spencer and Hochbaum.^33,34^ To our knowledge, the design of conditional heteromeric assemblies of >4 helices has yet to be achieved. Thus, we set out to design a heterohexameric, A_3_B_3_-type αHB, for which the individual helices were unfolded in solution and coassembled when mixed. As detailed below, our starting point was a de novo-designed all-parallel, homohexameric αHB, CC-Hex2, for which we have sequence-to-structure relationships and an X-ray crystal structure.^8^

## Materials and Methods

### General

All reagents were purchased from Sigma-Aldrich (Gillingham, UK), Fisher Scientific (Loughborough, UK), or Merck (Darmstadt, Germany) and used without further purification. All Fmoc-protected amino acids were purchased from either Sigma-Aldrich or Fluorochem. Biophysical data collection was typically carried out in HEPES-buffered saline (HBS, 25 mM HEPES, 100 mM NaCl, pH 7.5, water). When variations of these are used, they are explicitly stated. Isoelectric points, masses, and molar extinction coefficients at 280 nm (ε280 nm) for peptides were calculated using Innovagen’s peptide property calculator. Molar extinction coefficients for selected peptides at 214 nm (ε214 nm) were calculated individually based on their polypeptide sequence. Diagram representations of protein/peptide structures and computed structure models were all processed in ChimeraX molecular visualization software. All instances of water refer to ultrapure Milli-Q. All 96-well plates were dispensed using an Eppendorf epMotion 5070 liquid handling robot (Hamburg, Germany). Pure peptide stocks were stored at −20 °C. All heteromeric peptide combinations were mixed in a 1:1 ratio unless otherwise specified.

### Automated Fmoc Peptide Synthesis

Automated microwave solid-phase peptide synthesis (SPPS) was performed on a CEM Liberty Blue apparatus (Buckingham, UK) synthesizer with inline UV monitoring. Syntheses were performed on 0.1 mmol scales. The resin (Rink amide MBHA, 0.65 mmol/g loading, 100–200 mesh) was weighed to enable synthesis on a 0.1 mmol scale. The peptide coupling reactions were performed by adding Fmoc-protected amino acids dissolved in dimethylformamide (DMF) (2.5 mL, 0.2 M), the coupling reagent *N,N*-diisopropylcarbodiimide (DIC) in DMF (1.0 mL, 1 M), and Oxyma Pure in DMF (1.0 mL, 0.5 M) to the respective resin. Standard couplings were performed at 90 °C for 4.5 min (100 W for 20 s, 60 W for 10 s, and 35 W for 240 s). Standard deprotections were performed using 20% (v/v) morpholine in DMF at 90 °C for 1.5 min (125 W 30 s, 32 W 60 s). All peptides were manually acetyl-capped through the addition of pyridine (0.5 mL) and acetic anhydride (0.25 mL) in DMF (9.25 mL), shaking at room temperature (rt) for 20 min. The resin was washed three time with DMF, followed by three times with dichloromethane (DCM) before cleavage. Peptides were cleaved from the resin with the addition of 5 mL of a mixture of 95:2.5:2.5 v/v trifluoroacetic acid (TFA)/H_2_O/triisopropylsilane (TIPS), shaking at room temperature for 3 h. The TFA solution was then filtered to remove the resin beads and was reduced in volume to ≈5 mL or lower using a flow of N_2_. Cleaved peptides were precipitated with cold diethyl ether (≈40 mL), isolated via centrifugation, and dissolved in a 1:1 mixture of acetonitrile (MeCN)/H_2_O. Crude peptides were lyophilized to yield a white or off-white powder.

### Semipreparative High-Performance Liquid Chromatography (HPLC)

All peptides were purified by reverse-phase HPLC on a JASCO apparatus fitted with pumps (PU-980), a degasser (DG-980–50), a UV/vis detector (UV-2077), and a column oven (CO-1560), controlled by an LC-NetII/ADC. HPLC was used with a Phenomenex Luna C18 (Macclesfield, UK) column (150 × 10 mm, 5 mm particle size, 100 Å pore size). Crude peptides were dissolved at 5 mg/mL in 20 or 40% v/v MeCN in H_2_O with 0.1% TFA, injected to the column, and eluted with a 3 mL/min linear gradient (20 or 40–100%) of MeCN in H_2_O with 0.1% TFA (pI > 7) or 25 mM ammonium bicarbonate (pI < 7), each over 30 min. Elution of the peptides was detected with inline UV monitoring at 220 and 280 nm wavelengths simultaneously. A column oven (50 °C) was employed to improve separation. Pure fractions were identified by analytical HPLC and matrix-assisted laser desorption/ionization–time-of-flight (MALDI-TOF) mass spectrometry, then pooled, and freeze-dried.

### Analytical HPLC

Analytical HPLC traces were obtained using a JASCO 2000 series HPLC system (Hachioji, Tokyo) and a Phenomenex Kinetex C18 (100 × 4.6 mm, 5 μm particle size, 100 Å pore size) column (Macclesfield, UK). Chromatograms were monitored at 220 and 280 nm wavelengths. The linear gradient matched that used for semipreparative HPLC but over 25 min at a flow rate of 1 mL/min. When required, a JASCO column oven (CO-1650) was used at 50 °C to assist peptide elution.

### Mass Spectrometry

Matrix-assisted laser desorption/ionization–time-of-flight (MALDI-TOF) mass spectra were collected on a Bruker UltrafleXtreme MALDI-TOF mass spectrometer (Coventry, UK) operating in positive-ion reflector mode. Peptides were spotted on a ground steel target plate using α-cyano-4-hydroxycinnamic acid dissolved in 1:1 MeCN/H_2_O as the matrix. Masses quoted are for the monoisotopic mass as the singly protonated species.

### Peptide Concentration Determination

Peptide concentration was determined at 280 nm using a Thermo Scientific Nanodrop 2000 (Waltham, USA) spectrometer or an Agilent (Stockport, UK) Cary 100 UV/vis spectrophotometer (ε_280_(Trp) = 5690 M^–1^ cm^–1^; ε_280_ (Tyr) = 1280 M^–1^ cm^–1^). For sequences without Trp or Tyr, concentrations were determined using the amide absorbance for each sequence, using values determined by Gruppen and Kuipers using the equation:^35^
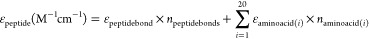
where ε_peptide bond_ is 923 M^–1^ cm^–1^, and ε_amino acid_ corresponds to the molar extinction coefficient of the free amino acid at 214 nm.

### Circular Dichroism (CD) Spectroscopy

Circular dichroism (CD) spectra were collected on JASCO (Hachioji, Tokyo) J-810 or J-815 spectrophotometers fitted with a Peltier temperature controller. Measurements were carried out in quartz cuvettes (Starna Scientific; Ilford, UK) with path length used dependent on peptide concentration (1 mm: >50 μM; 5 mm: 10–50 μM; 10 mm: <10 μM). The instrument was set with a sensitivity of 100 mdeg, a pitch and bandwidth of 1 nm, and a scan rate of 100 nm.min^–1^ at 20 °C with a response time of 1 s. Readings were averaged over eight accumulative scans. Readings were taken from 200–260 nm. Peptide samples were made up in HBS. Baseline recordings using the same buffer, cuvette, and instrument parameters were subtracted from each spectrum.

Temperature-variable scans were taken at 222 nm, ranging from 5 to 95 °C at a data pitch of 1 °C with a 16 s delay and a temperature ramping rate dependent on the path length (1 mm: 60 °C h^–1^, 5 mm: 45 °C h^–1^, 10 mm: 30 °C h^–1^).

To standardize the data, each spectrum was converted from ellipticities (mdeg) to mean residue ellipticities (MRE, deg cm^2^ dmol^–1^ res^–1^) by normalizing for the concentration of peptide bonds and the cuvette path lengths:
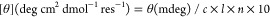
where the variable θ is the measured difference in absorbed circularly polarized light in millidegrees, *c* is the μM concentration of the compound, *l* is the path length of the cuvette in mm, and *n* is the number of amide bonds in the polypeptide, including the *N*-terminal acetyl group.

### Analytical Ultracentrifugation

Analytical ultracentrifugation sedimentation velocity (SV) was completed at 20 °C in either a Beckman Optima XL-A or XL-I analytical ultracentrifuge equipped with an An-50 Ti rotor. SV cells were constructed with either aluminum or Epon 2-channel centerpieces and quartz windows (Beckman Coulter; High Wycombe, UK). Sample channels were made up of 300 or 400 μL of sample for Epon and aluminum cells. Samples consisted of 150 μM peptide in HBS. The reference channels (Epon, aluminum) contained 310 or 410 μL buffer. Samples were centrifuged at 50,000 rpm, with absorbance scans (280 nm) taken across a radial range of 5.8–7.3 cm at 5 min intervals after an initial 5 min delay for 120 scans. The data acquired were fitted to a continuous c(s) distribution model using SEDFIT^36^ with 95% confidence limits. The baseline, bottom, frictional coefficient (*f*/*f*_0_), and systematic time-invariant noise were fitted. SEDNTERP^36^ was used to calculate partial specific volumes (*v̅*) of peptides and buffer densities (ρ) and viscosities (η).

Analytical ultracentrifugation sedimentation equilibrium (SE) was conducted at 20 °C in a Beckman Optima XL-A or XL-I analytical ultracentrifuge equipped with an An-50 Ti rotor. The SE cells composed of a 6-channel Epon centerpiece with quartz windows. The sample channel was made up of 70 μM of peptide (35 μM each A and B peptides) in HBS. The reference channels had 120 μL of buffer. Absorbance scans (280 nm) were taken every 8 h, with a second scan to monitor the equilibrium state taken 1 h later, at speeds between 20,000 and 48,000 rpm. Data were fitted to a single ideal species model using SEDPHAT.^36^ Statistical analysis was carried out by a Monte Carlo analysis (1000 iterations, randomized start points, 95% confidence limits) of the acquired fits.

### DPH Binding Assay

All ligand-binding fluorescence experiments were recorded in a BMG Labtech (Aylesbury, UK) Clariostar plate reader at 25 °C. Binding experiments were completed with 1,6-diphenyl-1,3,5-hexatriene (DPH) at 1 μM in HBS and 5% v/v dimethyl sulfoxide (DMSO). Peptides were titrated into the DPH solutions at concentrations ranging 1–300 μM. These were then calculated into relative assembly concentration by dividing by the oligomeric state. Peptides and ligands were equilibrated for 2 h at rt with constant shaking. Fluorescence spectra were measured using excitation (λ_ex_) of 1,6-diphenyl-1,3,5-hexatriene (DPH) with emissions (λ_em_) at 455 nm (±10 nm). Dissociation constants (*K*_D_) were calculated by fitting a single-site binding model:
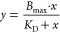
where *x* is the concentration of peptide, *B*_max_ is the fluorescence signal when all the constant component is bound, and *y* is the fraction of the bound component being monitored via the fluorescence signal.

### Fluorescence Quenching Experiments

Fluorescence quenching experiments were performed following the previously published procedure.^37^ Mixtures were prepared as an equimolar ratio of acidic and basic peptide analogues at 50 μM of the 4-cyanophenylalanine (4CF)-containing peptide. For both 4CF peptides, fluorescence was first tested in isolation before mixing with an equivalent concentration of MSe-containing peptides. Samples were prepared at room temperature and were also annealed at 95 °C for 60 s, then slowly cooled back to room temperature over 2 h. Fluorescence experiments were then conducted using a JASCO FP-6500 spectrofluorometer (Hachioji, Tokyo) and with either 26.50-F/Q/10 or 26.160-F/Q/10 quartz cuvettes provided by Starna Scientific (Hainault, UK). Samples were excited at 240 nm with a bandwidth of 3 nm, while readings were taken from 260 to 400 nm at a bandwidth of 1 nm reading 200 nm/min. A 1 s response time was used with a 0.5 nm data pitch. A manual voltage of 500 was used to amplify the signal. The experiments were conducted in 25 mM HEPES at pH 7.5 in the absence of NaCl as chloride ions are known to quench 4CF fluorescence.^37^

### Crystal Growth

Diffraction-quality crystals were grown using a sitting-drop vapor-diffusion method. Freeze-dried peptides were resuspended in deionized water to concentrations of 10 mg mL^–1^. CC-Hex2-A-3-***g*** and CC-Hex2-B-3-***g*** were mixed in a 1:1 ratio and annealed by heating to 95 °C and slowly cooling back to room temperature. Commercially available sparse matrix screens were used (JCSG-plusTM, Structure Screen 1 + 2, ProPlexTM, Morpheus, and PACT PremierTM), and the drops were dispensed using a robot (Oryx8; Douglas Instruments). For each well of an MRC 2 drop plate, 0.3 μL of the peptide solution was equilibrated with 0.3 μL of the screen solution in parallel with 0.4 μL of the peptide solution equilibrated with 0.2 μL of the screen solution, and the plate was incubated at 20 °C. To aid with cryoprotection, crystals were soaked in their respective reservoir solutions containing 25% glycerol prior to freezing.

### X-ray Crystal Structure Determination

X-ray diffraction data were collected at Diamond Light Source (Didcot, UK) on beamline I24 (Supplementary Table 2). Data were processed using the automatedXia2 pipeline, which ports data through DIALS (2.0.2) to POINTLESS (1.11.1) and AIMLESS (0.5.32), as implemented in the CCP4 suite. The structure was phased using ab initio phasing using ARCIMBOLDO_LITE. The initial phases were modeled into and refined using BUCCANEER. The final structure was obtained after iterative rounds of model building with COOT and refinement with REFMAC5 (7.1) and Phenix Refine (1.19.2_4158). TLS parameters were used during refinement as one group per chain for all structures. Torsion NCS restraints were used for fragments with <2 Å RMSD and 90% sequence identity. Solvent-exposed atoms lacking map density were either deleted or left at full occupancy. Data collection and refinement statistics are provided in Supplementary Table 2.

### Multimer Structure Prediction

AlphaFold-Multimer (AF-M) predictions were generated using ColabFold (version 1.5.2) without MSAs.^38−40^ 100 seeds for all five models were sampled, generating 500 predictions per sequence. Amber was used to minimize the top five ranking predictions on a NVIDIA RTX A5500. All other parameters for AF-M were kept at default values and are shown in the JSON configuration below:
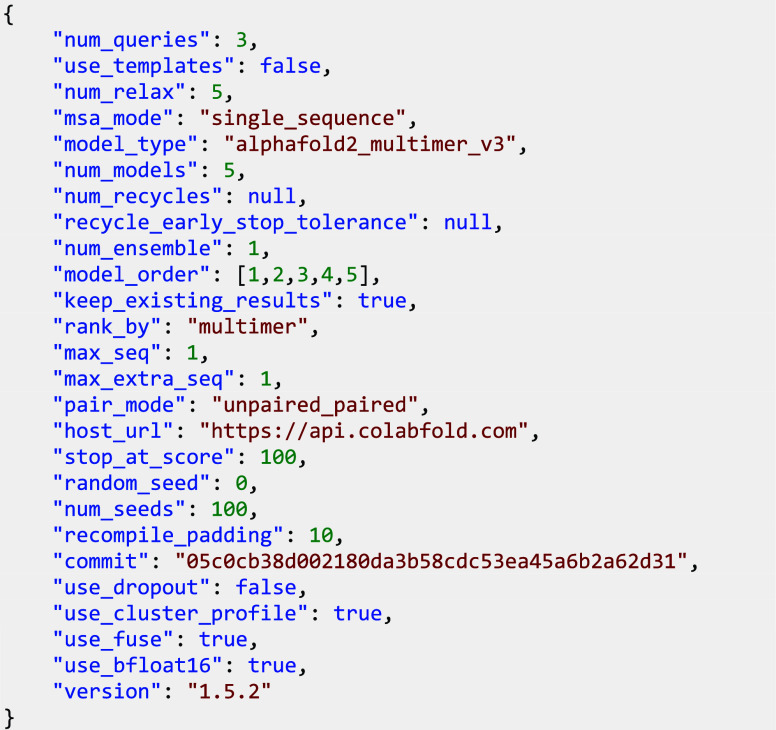


The CC-Hex2-AB-3-***g*** computed structure models were clustered based on structure similarity using template modeling scores (TMscores).^41^ TMscores were subsequently normalized for fraction aligned (normalized TMscore = TMscore × Fraction aligned) to punish incomplete alignments. Results were binned into clusters with 0.95 Normalized TMscore similarity.

AlphaFold3 predictions were run using the public Web server. For analysis, the predicted structure with the best ranking_score is used. All predictions were run with seed 42.

## Results and Discussion

The first aim of this study was to generate pairs of peptides that were unfolded in solution individually but assembled into a heterohexameric α-helical barrel (αHB) when mixed. Therefore, as a starting point, we took the CC-Hex2 peptide sequence, which forms a parallel homohexameric αΗΒ in solution and the crystal state.^8^ This peptide has four heptad repeats starting at a ***c*** position of the heptad repeat, and the ***g***, ***a***, ***d***, and ***e*** sites are occupied by serine (Ser, S), leucine (Leu, L), isoleucine (Ile, I), and alanine (Ala, A), respectively, which define the interhelical contacts and specify oligomeric state. Complementary charged glutamic acid (Glu, E) and lysine (Lys, K) residues at the flanking ***c*** and ***b*** sites, respectively, contribute to the helix–helix interactions through potential salt-bridge formation (Figure 1B).^8^ To design CC-Hex2-A_3_B_3_, we kept the *g***-***a***-***d***-***e* signature and altered the peripheral ***b*** and ***c*** sites to generate acidic (A) and basic (B) peptides: in CC-Hex2–4-A, ***b*** = ***c*** = Glu; and in CC-Hex2–4-B, ***b*** = ***c*** = Lys.^4^ The remaining ***f*** positions were made to be glutamine (Gln, Q), Lys, and tryptophan (Trp, W) or tyrosine (Tyr, Y) for solubility and to add chromophores (Table 1). The 4-heptad peptides (Table S1) were made by solid-phase peptide synthesis (SPPS), and their identity was confirmed by MALDI-TOF mass spectrometry (Figures S1 and S2).

**Table 1:**
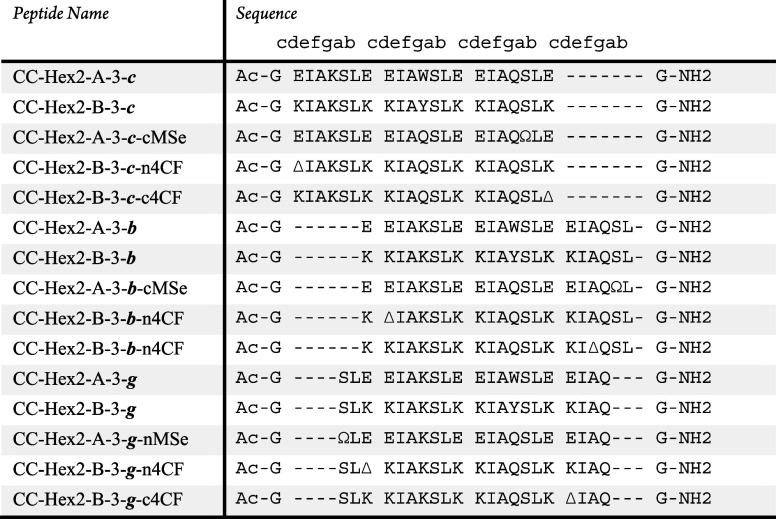
Designed Sequences for the Peptides Designed in This Study^a^

a Δ represents 4-cyanophenylalanine (4CF), and Ω denotes selenomethionine (MSe). Ac- denotes acetylated N terminal, −NH_2_ C terminal amide groups.

To determine whether the peptides assembled conditionally, first we used circular dichroism (CD) spectroscopy to assess the secondary structure of the peptides in aqueous buffer at neutral pH (Figure S3). However, it quickly became apparent that our initial sequences did not meet the first of the design criteria, as the individual peptides formed highly α-helical structures and, moreover, precipitated when the anionic and cationic peptides were mixed. We have observed similar behavior with heterotetramers^10^ and suggest that the highly and oppositely charged homomers form aggregates.

**Figure 2:**
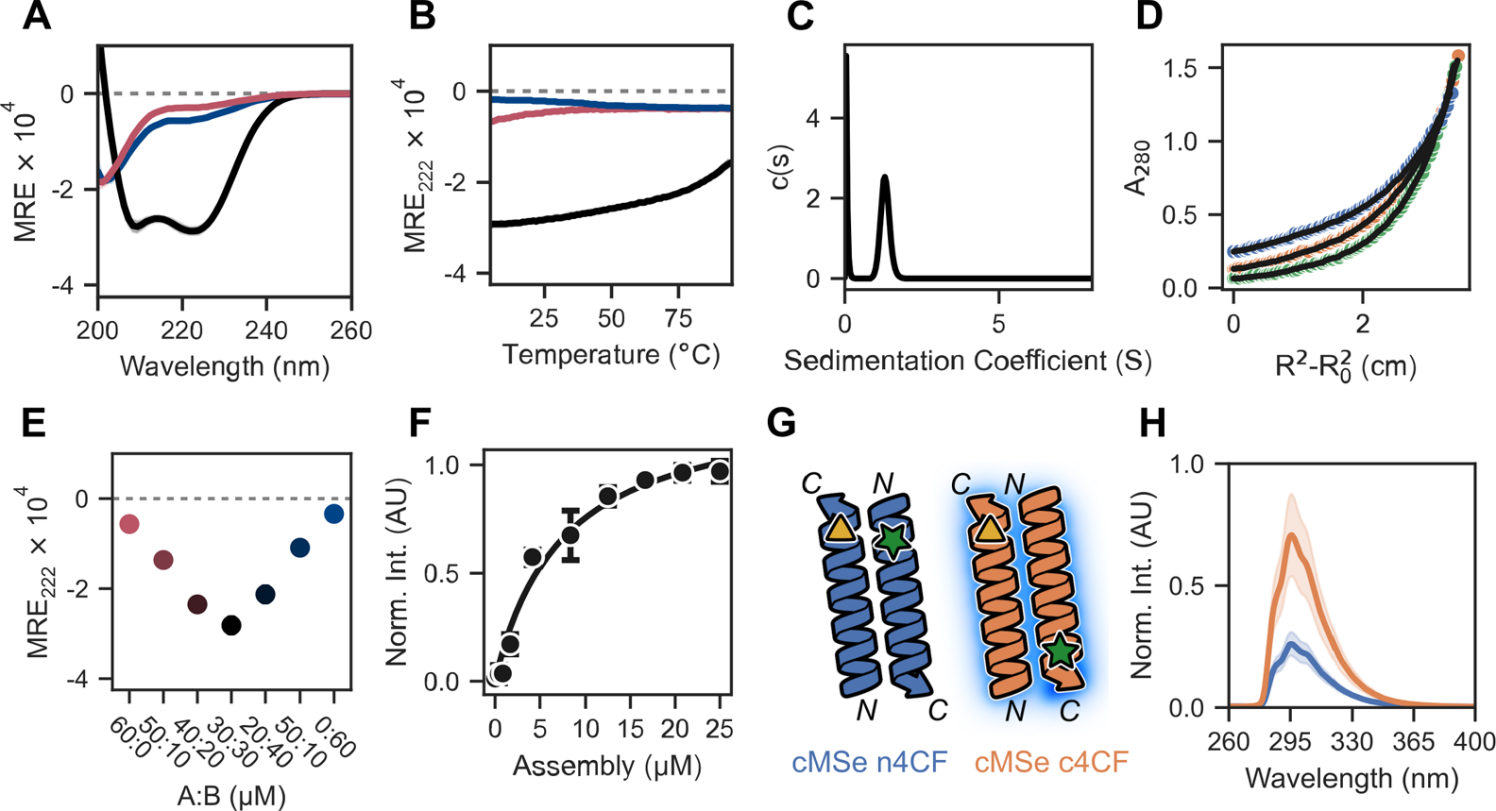
Biophysical characterization of CC-Hex2-AB-3-***c***. (A, B) CD spectra at 20 °C (A), and temperature-dependent CD signals monitored at 222 nm (B) for CC-Hex2-A-3-***c*** (red), CC-Hex2-B-3-***c*** (blue), and the mixture (black). (C) Analytical ultracentrifugation sedimentation velocity c(s) distribution fit recorded at 20 °C, 280 nm and 50 krpm (*v̅* = 0.765 cm^3^ g^–1^; s = 1.256 S; s20w = 1.316 S; *f*/*f*_0_ = 1.2). (D) Analytical ultracentrifugation sedimentation equilibrium data for CC-Hex2-AB-3-***c*** (fitted molecular weight = 15206 Da (6.0 × monomer mass), and 95% confidence limits = 14941–15502 Da. Speeds: 25 (blue), 35 (orange), and 45 (green) krpm. (E) Concentration-dependent CD signal monitored at 222 nm for 60 μM CC-Hex2-AB-3-***c*** with different compositions of CC-Hex2-A-3-***c*** and CC-Hex2-B-3-***c***. Markers show the mean of the data, and range bars represent one standard deviation; *N* = 3. (F) Saturation binding curves with DPH (1 μM); experimental data (filled circles) and fit (solid line) return *K*_D_ = 7.3 ± 0.2 μM (*R*^2^ = 0.981). Peptide concentration was converted to αHB concentration assuming an oligomeric state of 6. (G) Cartoon depicting possible orientations of adjacent A-B helices in the proposed antiparallel complexes, and their predicted effects on the proximity of the 4-cyanophenylalanine fluorophore (4CF, green star) and the selenomethionine quencher (MSe, yellow triangle). "n" and "c" indicate mutations near the N and C termini, respectively. Fluorescence is quenched if the pair are adjacent. (H) Fluorescence quenching assay for labeled CC-Hex2-AB-3-***c*** peptides. CC-Hex2-A-3-***c***-cMSe + CC-Hex2-B-3-***c***-n4CF (blue) is quenched relative to CC-Hex2-A-3-***c***-cMSe + CC-Hex2-B-3-***c***-c4CF (orange). Filled regions represent one standard deviation of the mean; *N* = 3.

To reduce homo-oligomerization of CC-Hex2-A and CC-Hex2-B, we removed a heptad repeat from each sequence to reduce the helix–helix hydrophobic interactions and hence the stabilities of the target and off-target assemblies (Table 1). We called these peptides CC-Hex2-A-3-***c*** and CC-Hex2-B-3-***c***; A and B for the acidic and basic chains, 3 for the number of heptads, and ***c*** for the starting register (Table 1 and Figures S1 and S2). In this case, CD spectroscopy showed that the individual peptides were largely unfolded but formed a soluble and highly α-helical assembly when mixed (Figure 2A). The unfolded nature of the individual peptides and stability of the mixture were confirmed by variable-temperature CD measurements, which showed that the signals for CC-Hex2-A-3-***c*** and CC-Hex2-B-3-***c*** changed little upon heating, but that for the mixture revealed a thermally stable complex with the start of an unfolding transition at ≈80 °C, as shown in Figure 2B.

Next, sedimentation velocity (SV) and sedimentation equilibrium (SE) analytical ultracentrifugation (AUC) experiments were used to probe the molecular weight of the A:B complex in solution. SV-AUC revealed a monodisperse oligomer of molecular weight ≈6 × the average monomeric masses of CC-Hex2-A-3-***c*** and CC-Hex2-B-3-***c*** (Figure 2C), which was confirmed by SE-AUC (Figure 2D). We then determined the stoichiometry of the complex by monitoring the α-helical CD signal at 222 nm at different compositions of CC-Hex2-A-3-***c*** and CC-Hex2-B-3-***c*** (Job plot; Figure 2E). This gave maximum signal at equimolar peptide concentrations, indicating a 1:1 stoichiometric complex. Combined, these data strongly indicated that we had made an A_3_B_3_ heterohexamer.

Coiled-coil assemblies above pentamer can form α-helical barrels with accessible central channels.^42^ To test whether we had made such a barrel—as opposed to a helical bundle with a consolidated hydrophobic core—the CC-Hex2-AB-3-***c*** assembly was tested with the environmentally sensitive dye, 1,3,5-diphenylhexatriene (DPH). When added to the peptide assembly, the dye fluoresced, and the concentration dependence of the response fitted to a single-site binding model, returning a *K*_D_ of ≈7 μM (Figure 2F). This binding constant is similar to that for CC-Hex2^42^ and, thus, is indicative of CC-Hex2-AB-3-***c*** forming an αHB.

Despite multiple attempts, we could not obtain diffraction-quality crystals for CC-Hex2-AB-3-***c*** mixtures needed for X-ray protein crystallography, and to determine directly whether the hexamer was parallel or antiparallel. Therefore, we used the proximity-based fluorescence quenching assay illustrated in Figure 2G.^10,37,43^ The quencher selenomethionine (MSe, Ω) was incorporated at the *C*-terminal ***g*** site of CC-Hex2-A-3-***c***, and the fluorescent 4-cyanophenylalanine (4CF, Δ) was placed at either the *N*-terminal ***c*** site or the *C*-terminal ***b*** site of CC-Hex2-B-3-***c*** (Table 1). Of note, early examples of incorporating aromatic residues at the interfacial positions of CCs led to bias toward antiparallel trimers;^44^ while this could also perturb the CC-Hex2-AB system, this is likely less of a concern given the polar nature of the unnatural 4CF side chain. If the peptides assembled into a parallel structure, like the parent CC-Hex2, the combination with both *C-*terminal substitutions would lead to quenching of 4CF fluorescence, and, conversely, the *C-* plus *N-*combination would fluoresce. However, we found that the latter—i.e., the combination of the *C-*terminal quencher and the *N-*terminal fluorophore—quenched the fluorescence (Figure 2G,H). This indicated that the CC-Hex2-AB-3-***c*** complex was most likely an antiparallel assembly in solution.

**Figure 3:**
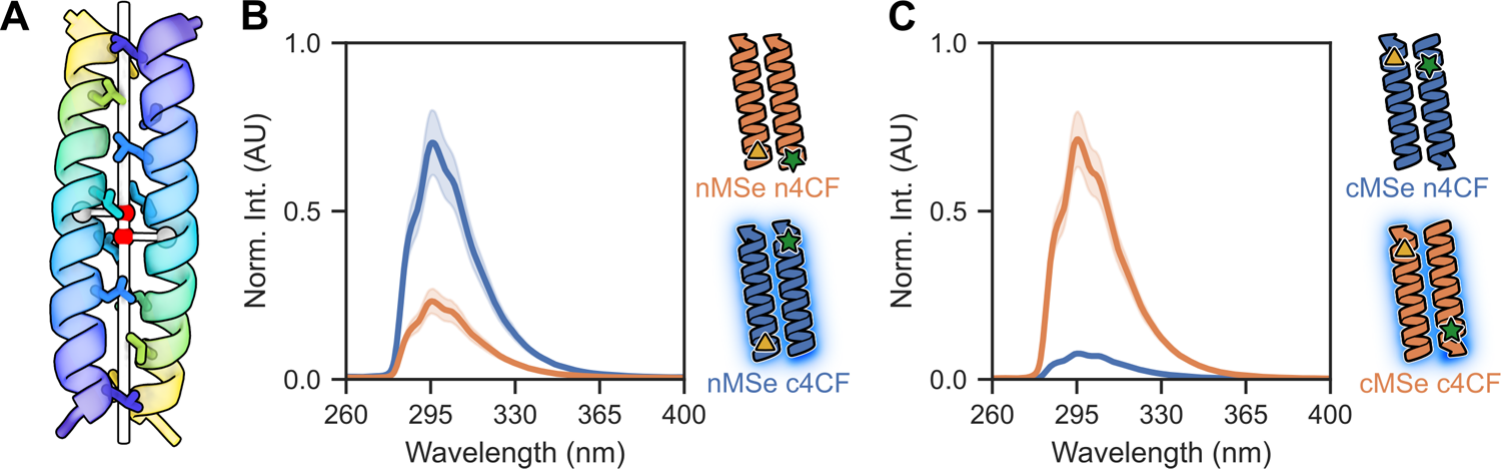
Manipulating helix orientation. (A) Antiparallel coiled coils slide along their *Z*-axes to facilitate interdigitation of side chains and KIH packing (sticks). The *Z*-shift is calculated as the distance between the projections of the centroids of each helix (gray spheres) onto the super helical axis (red spheres). For demonstration, an antiparallel dimer is shown (PDB ID: 7Q1T). (B, C) Fluorescence quenching assays for labeled CC-Hex2-AB-3-***g*** (B) and CC-Hex2-AB-3-***b*** (C) peptides. The colored labels denote the positions of the 4CF and MSe pairs at the “n” or “c” termini.

This result was surprising as we had based the heterohexamer design on the CC-Hex2 sequence, which forms an all-parallel α-helical assembly, confirmed by X-ray crystallography.^8^ However, it appears that, at least when reduced to three heptads in length and reconfigured into an A_3_B_3_ heteromeric design, specificity for parallel helix orientation is lost in favor of the antiparallel arrangement of helices (Figure 1B,C). While parallel CCs are largely *C_n_*-symmetric structures in which the helices and their registers are fully aligned—i.e., the equivalent ***a*** sites of all helices are at the same level along the central *Z*-axis—many antiparallel structures have a helical offset (*Z*-shift) between adjacent helices (Figure 3A).^45^ This offset is believed to help with side-chain interdigitation to achieve better KIH packing of coiled coils.^5,45−48^ In turn, this takes the sequences of adjacent helices out of alignment; i.e., ***a*** site Cα atoms do not align with ***a*** site Cα atoms of adjacent helices, and so on.

On this basis, we wondered if the starting register of coiled-coil regions in peptide sequences—i.e., ***a***, ***b***, ***c***, ***d***, ***e***, ***f***, or ***g***—might influence whether a *Z*-shift was accessible or not and hence the balance between parallel and antiparallel arrangements of helices. Therefore, to explore this, we synthesized peptides in the other six registers, i.e., CC-Hex2-AB-3-***a***, *-****b***, -***d***,-***e***, -***f***, and -***g*** and tested them experimentally (Tables 1, S1 and S2).

CD spectroscopy (Figures S3 and S4) and AUC experiments (Figures S9 and S10) were conducted for all new peptide variants (Table 1). Like the parent CC-Hex2-AB-3-***c***, CD spectroscopy showed that for CC-Hex2-AB-3-***a***, -***b***, -***d***, and -***g***, the individual A and B peptides were unfolded but when mixed formed soluble and highly helical assemblies. In contrast, CC-Hex2-AB-3-***e*** and ***f*** aggregated and precipitated when the A and B peptides were mixed, suggesting the formation of aggregates rather than discrete assemblies. These were not pursued further in this study. As with CC-Hex2-AB-3-***c****,* CC-Hex2-AB-3-***a***, *-****b***, **-*d***, and *-****g*** were thermally stable and showed the beginnings of unfolding transitions from ≈80 °C. In SV-AUC and SE-AUC experiments, CC-Hex2-AB-3-***a*** and -***d*** gave polydisperse assemblies ranging from 5 to 12 × the average monomeric masses. Therefore, these two systems were not explored further. AUC showed that both CC-Hex2-AB-3-***b*** and -***g*** assemble into monodisperse hexamers. Concentration-dependent CD experiments confirmed A_3_:B_3_ stoichiometry (Figures S6 and S8), and both bound DPH with low μM affinities (Figure S11).

Next, we synthesized the chromophore- and quencher-labeled variants of the -***b*** and -***g*** pairs (Table 1, Figure 3) and mixed the different peptide combinations as described for CC-Hex2-AB-3-***c***. CC-Hex2-AB-3-***g*** showed reduced fluorescence when the substituted amino acids were positioned at the same terminus (*N-* + *N-*), suggesting an assembly with a parallel helix orientation (Figure 3B). In contrast, CC-Hex2-AB-3-***b*** showed reduced fluorescence only when the residues were placed at different termini (*C-* + *N-*, Figure 3C), suggesting an antiparallel assembly, like CC-Hex2-AB-3-***c***.

**Figure 4:**
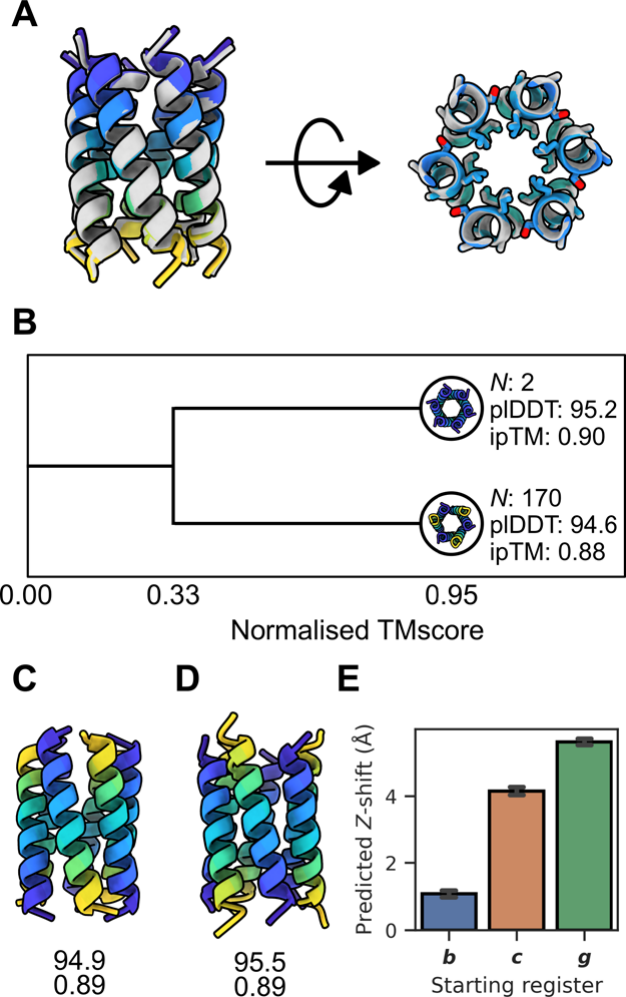
Structural features of CC-Hex2-AB-3-***b***, **-*c***, -***g***. (A) 1.9 Å X-ray crystal structure of CC-Hex2-AB-3-***g*** (PDB ID: 9EVG, gray) aligned to its top ranked computed structure model (colored, Cα-RMSD: 0.52 Å). Left, side on view of the all-parallel arrangement shows a *Z*-shift near 0 Å between helices. Right, knob-into-hole packing. For clarity, only the Cα–Cβ bonds are shown for side chains at the ***b***, ***c***, and ***f*** positions. (B) Clustering of the best AF-M predictions (ipTM > 0.7). Clustered by normalized TMscore (structure similarity = 0.95). Two clusters appear, representing the parallel and antiparallel predictions. Representatives are taken as the model with the highest confidence metrics. *N* denotes cluster size. Bottom, top-ranked computed model of CC-Hex2-AB-3-***b*** (C) and CC-Hex2-AB-3-***c*** (D). Numbers denote plDDT (top) and ipTM (bottom). (E) AF-M computed structure model predicted *Z*-shift between helices of antiparallel poses.

With a full set of solution-phase data for the two new peptide assemblies, we sought to determine X-ray structures for CC-Hex2-AB-3-***b*** and CC-Hex2-AB-3-***g***. Only CC-Hex2-AB-3-***g*** generated a usable diffraction pattern. The 1.9 Å resolution structure revealed a parallel heterohexameric coiled-coil barrel with alternating A and B chains (Figure 4A, Table S3), which is completely consistent with the solution-phase data.

In lieu of experimental atomic-resolution structures for CC-Hex2-AB-3-***b*** and ***c***, we computed structural models with AlphaFold-MultimerV3 (AF-M) implemented in ColabFold.^39,40^ First, we tested its capability to predict the CC-Hex2-AB-3-***g*** structure. Initially, the default configuration of ColabFold was used, which only sampled a small degree of model diversity with one seed used for each of the five AF-M models, creating a total of five predictions. With this limited sampling, the top prediction was highly confident (ipTM > 0.7), but it did not match the crystal structure; the experimental structure is all parallel, but the model predicted antiparallel helices (Figure 4A). Others have shown that more extensive sampling may be required to correctly predict multimeric structures.^40,49^ Therefore, we increased sampling through additional random seeds. This alters the stochastic elements of the model, which can lead to more model diversity and consequently better predictions. We increased the number of random seeds from 1 to 100, generating 100 predictions per model for a total of 500 predictions per sequence. To observe the structural diversity of the predictions, high-confidence results (ipTM > 0.7, *N* = 172) were clustered by structural similarity using a normalized TMscore (TMscore × fraction aligned, Figure 4B).^41^ This gave two groups when clustered by a structure similarity of 0.95 with all-parallel (*N* = 2) and antiparallel (*N* = 170) arrangements of helices. Thus, overall, the AlphaFold predictions are biased toward the incorrect antiparallel models by 85:1, which is concerning. That said, the two highest-confidence predictions were for the parallel models (Figure 4B). Indeed, these were near-superposable with the X-ray crystal structure of CC-Hex2-AB-3-***g*** (Cα-RMSD = 0.52 Å, Figure 4A).

Mindful of the caveat in this approach, we applied it to generate models for CC-Hex2-AB-3-***b*** and ***c***, again using 100 seeds across the five different models for 500 total predictions each. The ratios of predicted models with all-parallel and with antiparallel arrangements of helices were 1:301 and 1:115, respectively. However, the top ranked predictions for both sequences were antiparallel 6-helix barrels with alternating A and B chains consistent with the solution-phase data (Figure 4C,D). On this basis, we assumed that these top predicted models for CC-Hex2-AB-3-***b*** and CC-Hex2-AB-3-***c*** might best represent the actual structures. With these models and the “theoretical”, but not experimentally observed, model of the antiparallel state of CC-Hex2-AB-3-***g***, we examined the hypothesis of *Z*-shift driving helix orientation. Z-shifts of the high-confidence models (ipTM > 0.7) for the three registers were calculated (Figure 4E). Interestingly, these were in the order ***g*** > ***c*** > ***b***. Moreover, at 5.6 Å, the *Z*-shift for the ***-g*** peptide is a whole α-helical turn (5.4 Å). Reasoning that larger *Z*-shifts lead to larger helical overhangs and less helix–helix contacts in the assembled CCs, it stands to reason that the antiparallel arrangement for the ***g**-*register peptide would be the least stable of these and less stable than the observed parallel structure. This analysis suggests a *Z*-shift threshold for the CC-Hex2-AB-3 designs to form antiparallel assemblies of ≈ 4 Å, and above this, the parallel state is favored. Although our analysis is limited and focused, it fits with larger analyses of experimental structures of parallel and antiparallel CCs from the RCSB PDB, albeit for smaller oligomers.^45,50^ From these studies, parallel CCs have *Z*-shift values (referred to as Δ*Z* and axial shift and defined slightly differently in those papers) are sharply centered around 0; whereas in antiparallel structures, they are ≈2 to 3 Å.

## Conclusions

Through a combination of rational peptide design and empirical redesign, we have delivered three heterohexameric A_3_B_3_-type α-helical barrel assemblies (αHBs). One of these, CC-Hex2-AB-3-***g***, has all-parallel helices, as is confirmed by solution-phase spectroscopic data and an X-ray peptide crystal structure. The data for the other two, CC-Hex2-AB-3-***b*** and CC-Hex2-AB-3-***c***, are consistent with antiparallel arrangements of helices in solution, but we could not confirm this by X-ray crystallography. We find that the sequences can be modeled as the parallel and antiparallel states by AlphaFold-Multimer (AF-M). However, this requires larger sampling than offered by the default AlphaFold settings and then close inspection of the models and prediction metrics. This is because, at least for our system, AF-M appears biased to predict antiparallel helix orientations. This is opposite to other observations for lower oligomeric state coiled coils (<5), which report a bias for parallel predictions.^51^

The serendipitous discovery that CC-Hex2-AB-3-***c*** forms an antiparallel assembly led to the hypothesis that the starting register of the coiled-coil sequence in peptide designs could contribute to specifying coiled-coil topology, (i.e., parallel, antiparallel, or mixed arrangements of helices). The reasoning is that different starting registers would lead to different helical overhangs for essentially the same knobs-into-hole packing between helices; and the longer the overhang, the less stable the antiparallel state relative to the parallel state, where the helices are usually aligned and do not overhang. To explore this, we examine the *Z*-shift of AF-M models for the antiparallel states of the ***b**-*, ***c**-*, and ***g**-*register peptides for which we have experimental data. From this, the antiparallel model for CC-Hex2-AB-***g*** has the largest predicted *Z*-shift, and this is outside the range observed across structurally defined natural CCs, albeit of lower oligomeric states.^45,50^ This is consistent with that peptide forming a parallel rather than an antiparallel assembly. This adds another design parameter that should be considered for controlling helix orientation in de novo coiled-coil peptides assemblies and proteins.

This work adds to existing literature on understanding coiled-coil peptide design and assembly.^10,33,34,52^ To date, the coiled-coil-design hierarchy has been: first, focus on residues at the largely hydrophobic, knobs-into-holes, helix–helix interfaces, as these have the most influence on coiled-coil stability and oligomeric state. Second, use interhelical salt-bridges to direct coiled-coil partnering, i.e., to form homo- or heteromeric assemblies. However, these can be used to steer parallel versus antiparallel helix orientations with what might be termed “classical” and “bar-magnet” charge patterning, respectively.^10,11^ Here, we add sequence register to the list of features that can influence coiled-coil assembly and helix orientation. However, we recognize that its contribution may be small. For example, here, we show that CC-Hex2-AB-3-***c*** forms antiparallel A_3_B_3_-type assembly in solution. Nonetheless, most previously reported de novo αHBs have ***c***-register and are parallel assemblies.^8,9^ Similarly, while CC-Hex2-AB-3-***g*** forms a parallel A_3_B_3_ αHB, the antiparallel tetramer, ap-CC-Tet, also has a ***g***-register but with bar-magnet charge patterning to drive the antiparallel state.^10^ Nonetheless, we suggest that the starting and ending point of the heptad registry and the corresponding helical overhangs should be considered as a variable when assessing new coiled-coil-based peptide and protein designs, as its subtle effects may be important particularly as peptide and protein design move on from targeting static structures to more-dynamic and functional assemblies.

## Data Availability

All data are available via the Zenodo Repository (https://zenodo.org/records/14616658). Scripts for data generation and visualization can be found on GitHub (https://github.com/woolfson-group/A3B3_paper_code).

## Supporting Information Available

Biophysical characterization, aHPLC, and MS for all peptides investigated in study (PDF)

## Accession Codes

CC-Hex2-AB-3-***c***, PDB ID: 9EVG.
