# Supplementary material for: De Novo Design of Parallel and Antiparallel A3B3 Heterohexameric α-Helical Barrels
Source: Biochemistry. 2025 Apr 14;64(9):1973–82. doi: 10.1021/acs.biochem.4c00584 (PMC12060282; doi:10.1021/acs.biochem.4c00584)
Supplement: Supplementary file 1 — bi4c00584_si_001.pdf [file bi4c00584_si_001.pdf]

Supporting Information for:

De novo design of parallel and antiparallel A_3B_3 heterohexameric α-helical barrels

Joel J. Chubb,[1,2†] Katherine I. Albanese,[1,4†] Alison Rodger,[3] and Derek N. Woolfson[1,4,5,6]*

[1]School of Chemistry, University of Bristol, Cantock's Close, Bristol BS8 1TS, UK

[2]School of Natural Sciences, Macquarie University, Sydney, New South Wales, 2019, Australia

[3]Research School of Chemistry, Australian National University, ACT, 2601, Australia

[4]Max Planck-Bristol Centre for Minimal Biology, University of Bristol, Cantock's Close, Bristol BS8 1TS, UK

[5]School of Biochemistry, University of Bristol, Medical Sciences Building, University Walk, Bristol BS8 1TD, UK

[6]Bristol BioDesign Institute, University of Bristol, Cantock's Close, Bristol BS8 1TS, UK

Table of Contents

1 Supplementary data 3

1.1 MALDI-TOF and analytical HPLC 7
1.2 Circular Dichroism (CD) spectroscopy 11
1.3 Analytical Ultracentrifugation (AUC) 14
1.4 Ligand Binding assays 15
1.5 AlphaFold3 computed structure models 16

1 SUPPLEMENTARY DATA

Table S1. MALDI-TOF and analytical ultracentrifugation statistics of designed peptides. *n.d.* not determined

Name	Monomeric mass expected (g/mol)	Monomeric mass observed MALDI-TOF (g/mol)	Molecular species observed MALDI-TOF	Partial specific volume ($\bar{v}$, $cm^3\ g^{-1}$)	Fitted mass AUC-SV (95 % confidence, 3SF, g/mol)	f/f0	s (S)	s20,w (S)	Fitted mass AUC-SE (95 % confidence, 3SF, g/mol)	AUC-SE molecular mass / monomer mass
CC-Hex2-A-4-***c***	3314.7	3316.6	$[M+H]^+$	n.d.						
CC-Hex2-B-4-***c***	3307.2	3308.4	$[M+H]^+$							
CC-Hex2-A-3-***a***	2543.8	2566.8	$[M+Na]^+$	0.765	17,200, 34,200	1.544, 1.544	1.273, 2.014	1.333, 2.110	n.d.	
CC-Hex2-B-3-***a***	2515.1	2516.0	$[M+H]^+$							
CC-Hex2-A-3-***b***	2543.8	2582.3	$[M+K]^+$	0.776	11,800	1.148	1.278	1.322	14,300	5.7
CC-Hex2-B-3-***b***	2515.1	2539.2	$[M+Na]^+$							
CC-Hex2-A-3-***b***-cMSe	2576.7	2600.4	$[M+Na]^+$	n.d.						
CC-Hex2-B-3-***b***-n4CF	2523.9	2547.1	$[M+Na]^+$							
CC-Hex2-B-3-***b***-c4CF	2587.1	2604.3	$[M+Na]^+$							
CC-Hex2-A-3-***c***	2543.8	2582.0	$[M+K]^+$	0.765	11,200	1.173	1.256	1.316	15,400	6.1
CC-Hex2-B-3-***c***	2515.1	2537.3	$[M+Na]^+$							
CC-Hex2-A-3-***c***-cMSe	2576.8	2601.2	$[M+Na]^+$	n.d.						
CC-Hex2-B-3-***c***-n4CF	2523.9	2524.7	$[M+H]^+$							
CC-Hex2-B-3-***c***-c4CF	2524.0	2524.7	$[M+H]^+$							
CC-Hex2-A-3-***d***	2543.8	2567.2	$[M+Na]^+$	0.765	13,000, 31,033	1.103, 1.103	1.479, 2.640	1.549, 2.766	n.d.	
CC-Hex2-B-3-***d***	2515.1	2516.0	$[M+H]^+$							
CC-Hex2-A-3-***e***	2543.8	2582.0	$[M+K]^+$	n.d.						
CC-Hex2-B-3-***e***	2515.1	2537.7	$[M+Na]^+$							
CC-Hex2-A-3-***f***	2543.8	2566.9	$[M+Na]^+$	n.d.						
CC-Hex2-B-3-***f***	2515.1	2515.2	$[M+H]^+$							
CC-Hex2-A-3-***g***	2543.8	2568.5	$[M+Na]^+$	0.76	14,200	1.200	1.482	1.548	15,200	6.0
CC-Hex2-B-3-***g***	2515.1	2517.2	$[M+H]^+$							
CC-Hex2-A-3-***g***-nMSe	2576.8	2600.9	$[M+Na]^+$	n.d.						
CC-Hex2-B-3-***g***-n4CF	2523.9	2524.7	$[M+H]^+$							
CC-Hex2-B-3-***g***-c4CF	2524.0	2548.1	$[M+Na]^+$							

Table S2. Designed sequences for partially characterized peptides used in this study. Ac-denotes acetylated N terminal, -NH2 C terminal amide groups.

Peptide Name	Sequence<br>***cdefgab cdefgab cdefgab cdefgab***
CC-Hex2-A-3-***a***	Ac-G -----LE EIAKSLE EIAWSLE EIAQS-- G-NH2
CC-Hex2-B-3-***a***	Ac-G -----LK KIAKSLK KIAYSLK KIAQS-- G-NH2
CC-Hex2-A-3-***d***	Ac-G -IAKSLE EIAWSLE EIAQSLE E------ G-NH2
CC-Hex2-B-3-***d***	Ac-G -IAKSLK KIAYSLK KIAQSLK K------ G-NH2
CC-Hex2-A-3-***e***	Ac-G --AKSLE EIAWSLE EIAQSLE EI----- G-NH2
CC-Hex2-B-3-***e***	Ac-G --AKSLK KIAYSLK KIAQSLK KI----- G-NH2
CC-Hex2-A-3-***f***	Ac-G ---KSLE EIAWSLE EIAQSLE EIA---- G-NH2
CC-Hex2-B-3-***f***	Ac-G ---KSLK KIAYSLK KIAQSLK KIA---- G-NH2

Table S3. Crystal structure refinement statistics for CC-Hex2-AB-3-***g***.

	CC-Hex2-AB-3-***g***
**PDB ID**	9EVG
**Data Collection**	
Source	Diamond I24
Detector	PILATUS3 6M
Wavelength	0.98
Resolution range	47.31 - 1.903 (1.971 - 1.903)
Space group	P 1 21 1
Unit cell: *a, b, c (Å)*	32.6756 94.6118 51.1156
α, β, γ (°)	90 91.7522 90
Total reflections	321327 (31423)
Unique reflections	24363 (2402)
Multiplicity	13.2 (13.1)
Completeness (%)	99.86 (99.26)
Mean I/sigma(I)	11.57 (1.59)
Wilson B-factor	29.29
R-merge	0.114 (0.2568)
R-meas	0.1189 (0.2673)
R-pim	0.03329 (0.0735)
CC1/2	0.997 (0.975)
CC*	0.999 (0.994)
**Refinement**	
Reflections used in refinement	24349 (2402)
Reflections used for R-free	1134 (95)
R-work	0.1921 (0.2743)
R-free	0.2185 (0.3311)
CC(work)	0.956 (0.809)
CC(free)	0.937 (0.645)
Number of non-hydrogen atoms	2020
macromolecules	1796
ligands	121
solvent	147
Protein residues	272

RMS(bonds)	0.004
RMS(angles)	0.45
Ramachandran favored (%)	99.6
Ramachandran allowed (%)	0.4
Ramachandran outliers (%)	0
Rotamer outliers (%)	0
Clashscore	1.09
Average B-factor	72.69
macromolecules	71.58
ligands	93.15
solvent	75.63
Number of TLS groups	12

1.1 MALDI-TOF and analytical HPLC

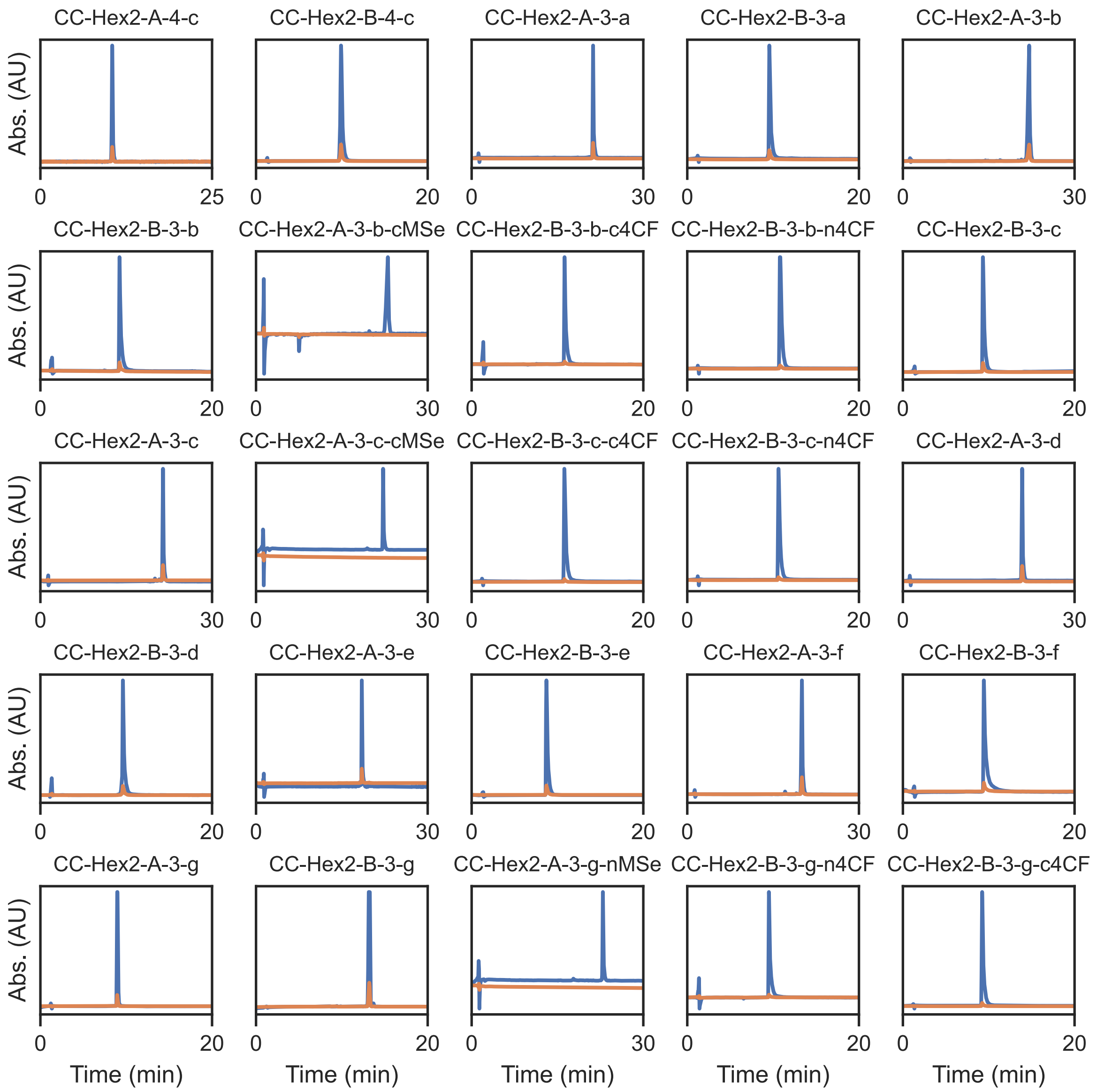

Figure S1. HPLC traces (blue 220, orange 280 nm) of peptides designed for this study. All traces are corrected for baseline absorbance.

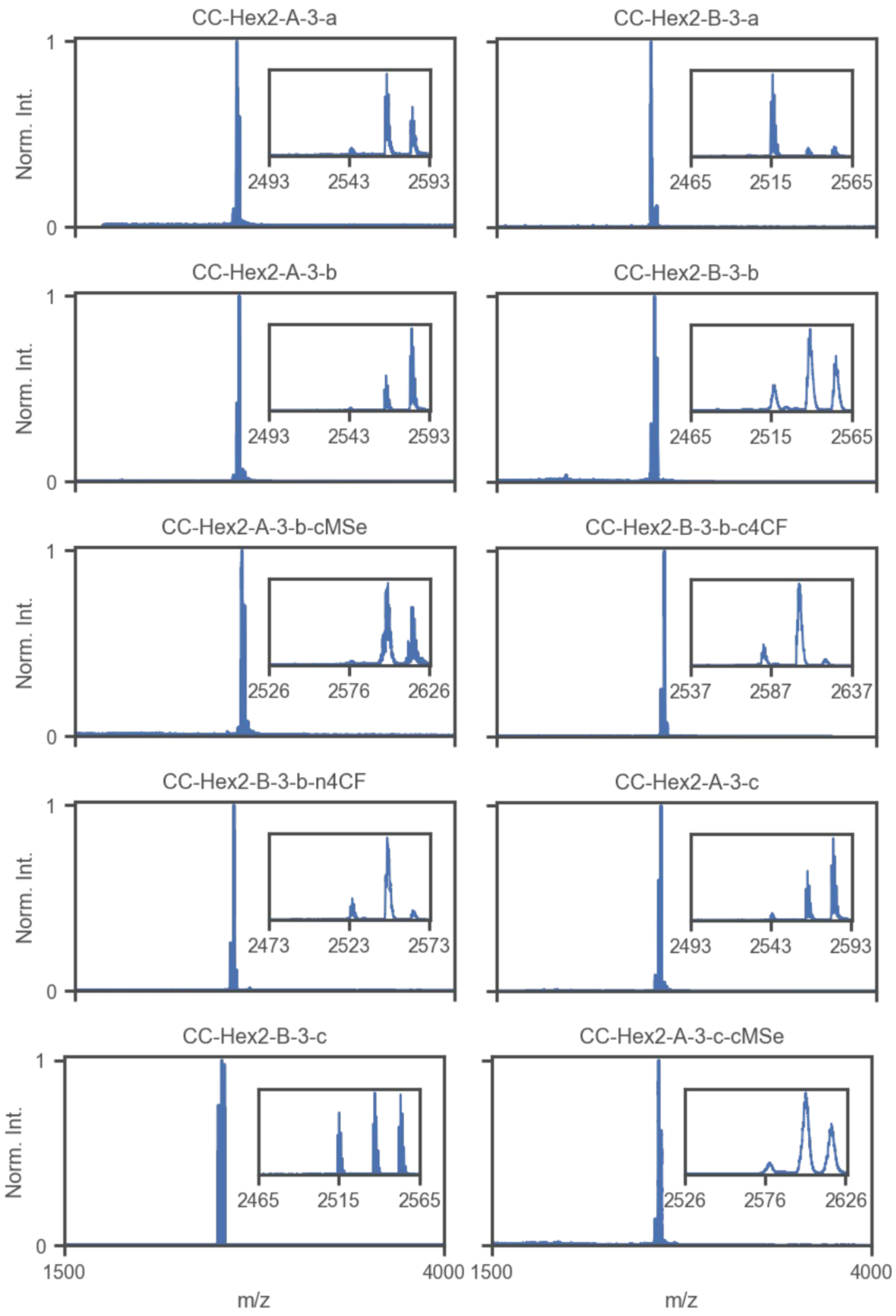
CC-Hex2-A-3-a
CC-Hex2-B-3-a
CC-Hex2-A-3-b
CC-Hex2-B-3-b
CC-Hex2-A-3-b-cMSe
CC-Hex2-B-3-b-c4CF
CC-Hex2-B-3-b-n4CF
CC-Hex2-A-3-c
CC-Hex2-B-3-c
CC-Hex2-A-3-c-cMSe
Norm. Int.
1
0
2493
2543
2593
2465
2515
2565
2526
2576
2626
2537
2587
2637
2473
2523
2573
1500
4000
m/z

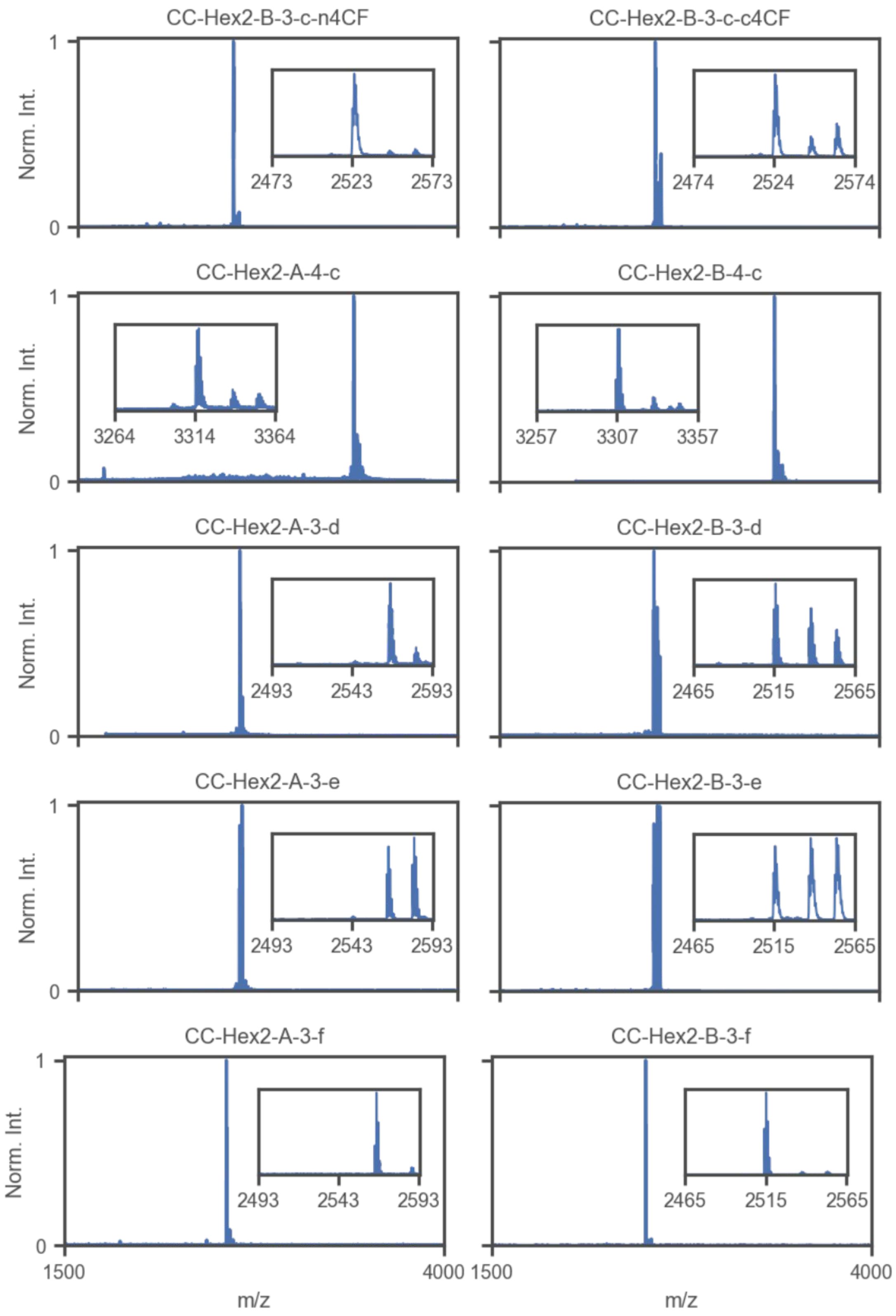
CC-Hex2-B-3-c-n4CF
CC-Hex2-B-3-c-c4CF
CC-Hex2-A-4-c
CC-Hex2-B-4-c
CC-Hex2-A-3-d
CC-Hex2-B-3-d
CC-Hex2-A-3-e
CC-Hex2-B-3-e
CC-Hex2-A-3-f
CC-Hex2-B-3-f
Norm. Int.
1
0
2473
2523
2573
2474
2524
2574
3264
3314
3364
3257
3307
3357
2493
2543
2593
2465
2515
2565
1500
4000
m/z

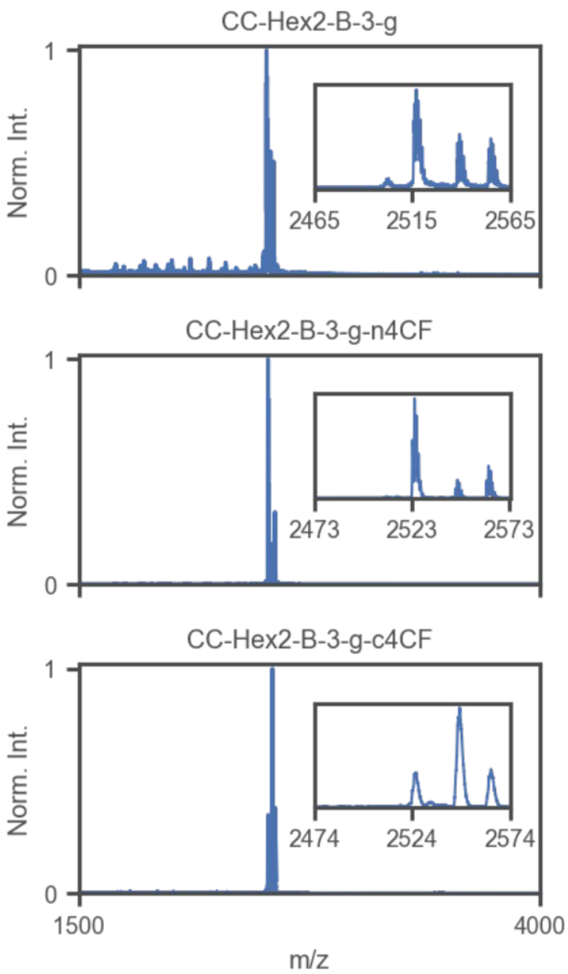

Figure S2. MADLI-TOF MS of peptides designed for this study. Observed masses and molecular species are shown in Table S1.

1.2 Circular Dichroism (CD) spectroscopy

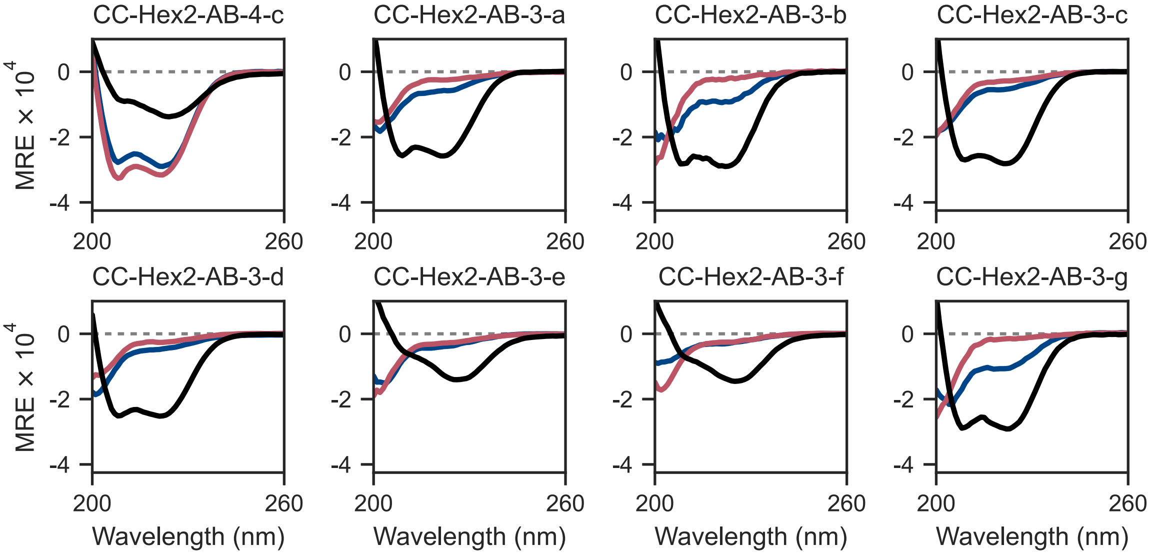

Figure S3. CD spectra at 20 °C for peptides designed for this study. Acidic peptides are shown in red, basic in blue and mixtures in black. MRE, mean residue ellipticity (deg cm2 $dmol^{-1}$ res^{-1}). Conditions: 100 μM peptide (50:50 when mixed), HBS.

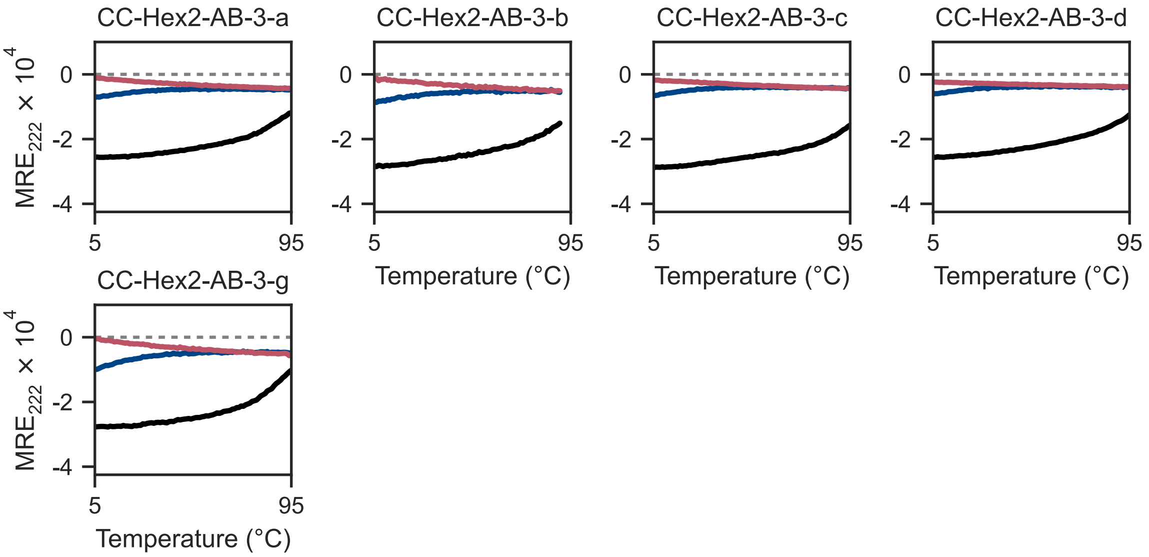

Figure S4. Temperature dependent CD signal, monitored at 222 nm for helical peptides designed for this study. Acidic peptides are shown in red, basic in blue and mixtures in black. MRE, mean residue ellipticity (deg cm2 $dmol^{-1}$ res^{-1}). Conditions: 100 μM peptide (50:50 when mixed), HBS.

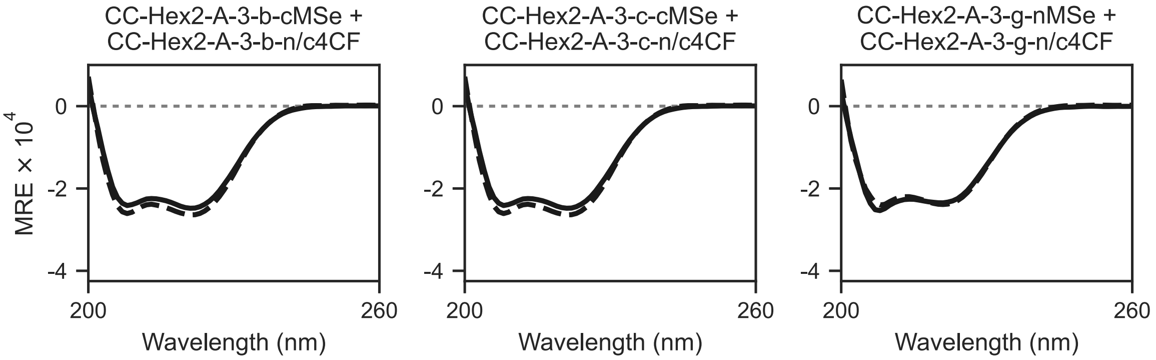

Figure S5. CD spectra at 20 °C for MSe and 4CF containing peptides designed for this study. Both combinations of 4CF variants are shown; CC-Hex2-A-3-***x***-n/cMSe + CC-Hex2-B-3-***x***-n4CF (solid), CC-Hex2-A-3-***x***-n/cMSe + CC-Hex2-B-3-***x***-c4CF (dashed), ***x*** denoting helix register (***b***/***c***/***g***). MRE, mean residue ellipticity (deg cm2 dmol^{-1} res^{-1}). Conditions: 100 μM peptide (50:50 when mixed), HBS.

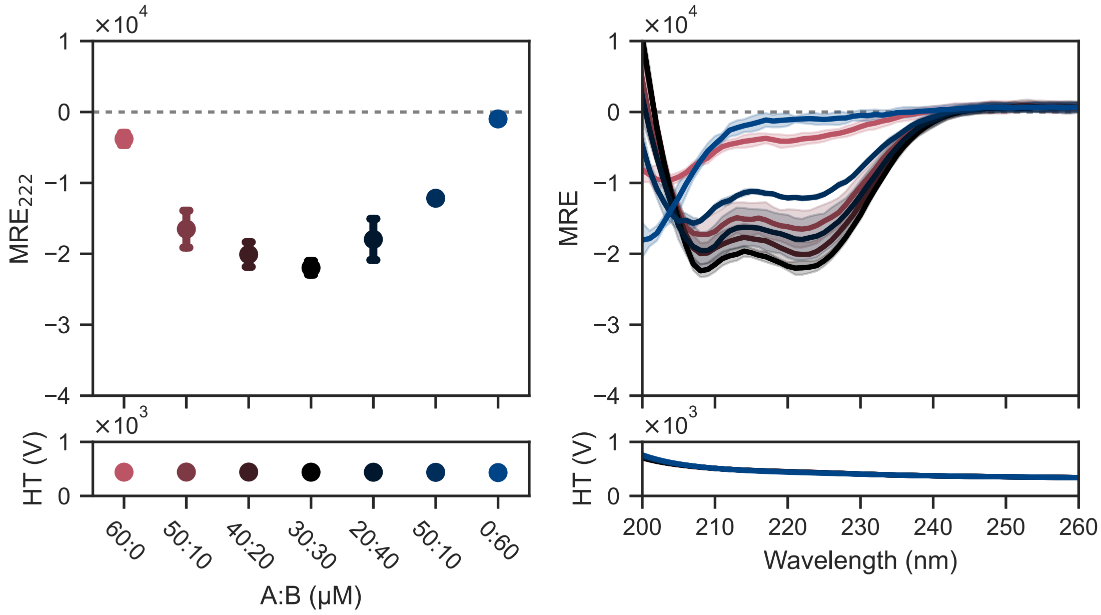

Figure S6. Concentration dependent CD signal monitored at 222 nm, (left) and CD spectra at 20 °C (right) for CC-Hex2-AB-3-b. Color denotes the ratio of CC-Hex2-A-3-b:CC-Hex2-B-3-b (red 1:0, black 1:1, blue 0:1). MRE, mean residue ellipticity (deg cm2 dmol-1 res-1). Conditions: HBS. Markers show the mean of the data and range bars represent one standard deviation of the mean. *N* = 3.

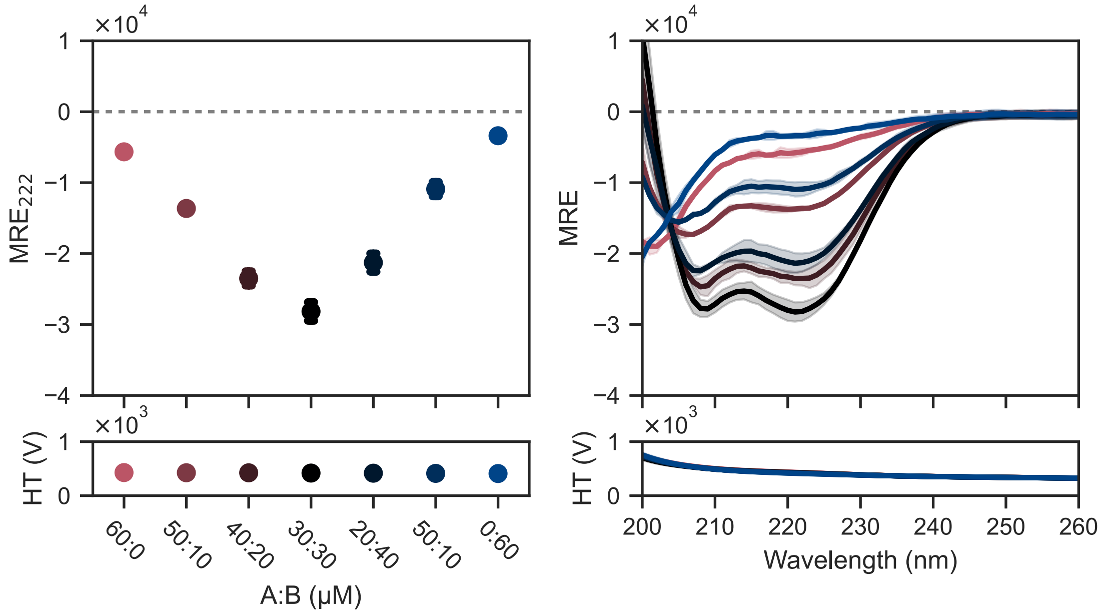

Figure S7. Concentration dependent CD signal monitored at 222 nm, (left) and CD spectra at 20 °C (right) for CC-Hex2-AB-3-c. Color denotes the ratio of CC-Hex2-A-3-c:CC-Hex2-B-3-c (red 1:0, black 1:1, blue 0:1). MRE, mean residue ellipticity (deg cm2 dmol-1 res-1). Conditions: HBS. Markers show the mean of the data and range bars represent one standard deviation of the mean. N = 2.

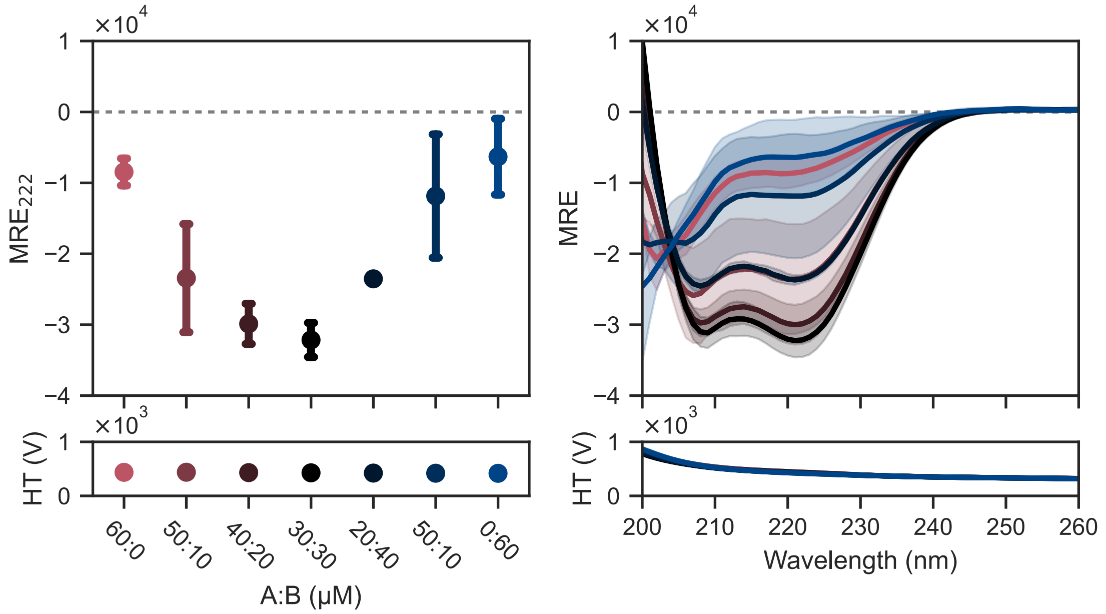

Figure S8. Concentration dependent CD signal monitored at 222 nm, (left) and CD spectra at 20 °C (right) for CC-Hex2-AB-3-g. Color denotes the ratio of CC-Hex2-A-3-g:CC-Hex2-B-3-g (red 1:0, black 1:1, blue 0:1). MRE, mean residue ellipticity (deg cm2 dmol-1 res-1). Conditions: HBS. Markers show the mean of the data and range bars represent one standard deviation of the mean. N = 3.

1.3 Analytical Ultracentrifugation (AUC)

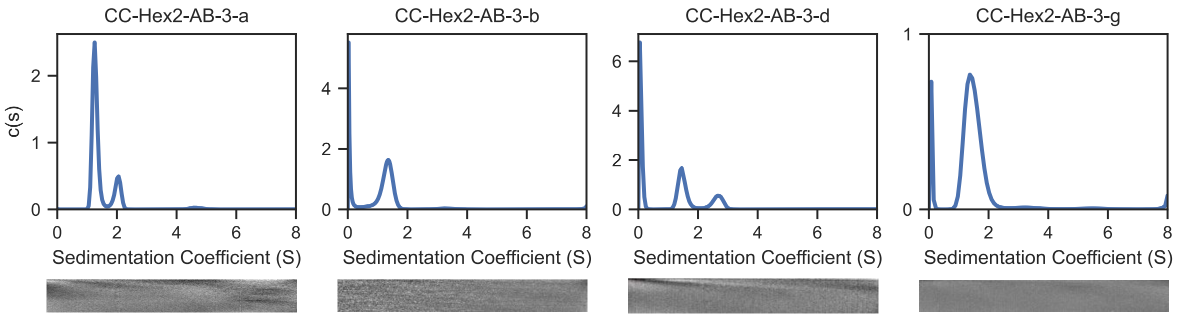

Figure S9. Analytical Ultracentrifugation sedimentation velocity traces of peptides designed for this study. Residuals are shown as a bitmap (below). Results for individual peptides can be found in Supplementary table 1. Conditions: 75 μM CC-Hex2-A-3-***x*** + 75 μM CC-Hex2-B-3-***x***, HBS.

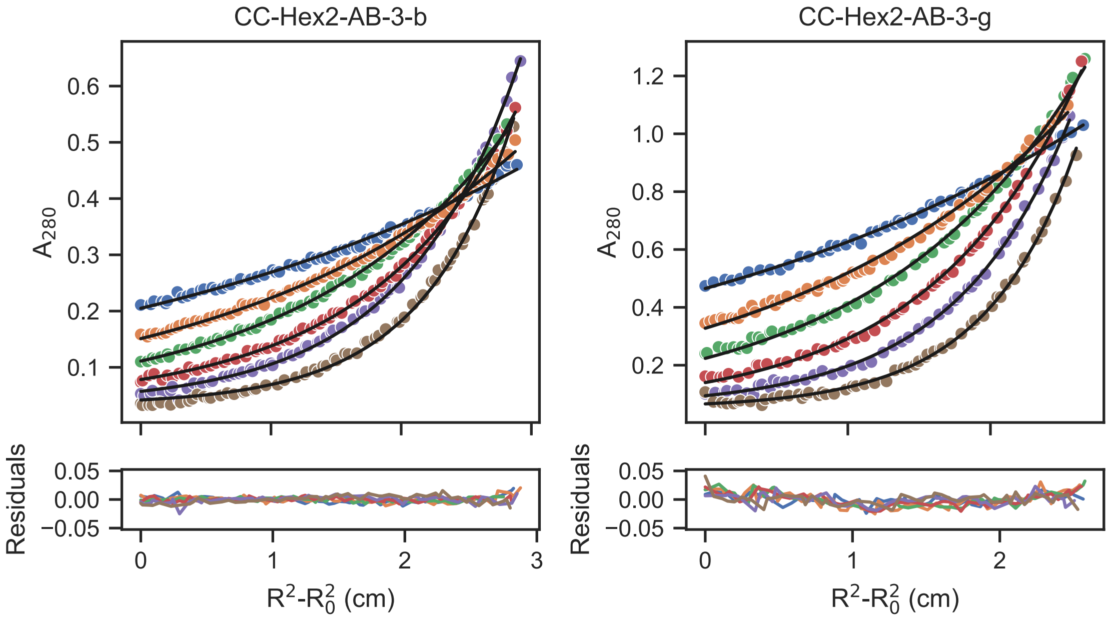

Figure S10. Analytical Ultracentrifugation sedimentation equilibrium traces of peptides designed for this study. Data (markers) and fit (solid lines) are shown in the upper plots with residuals shown below. Colors indicate rotor speed (rpm): 20,000 (blue), 25,000 (orange), 30,000 (green), 35,000 (red), 40,000 (purple), 45,000 (brown). Results for individual peptides can be found in Supplementary table 1. Conditions: 35 μM CC-Hex2-A-3-***x*** + 35 μM CC-Hex2-B-3-***x***, HBS.

1.4 Ligand Binding assays

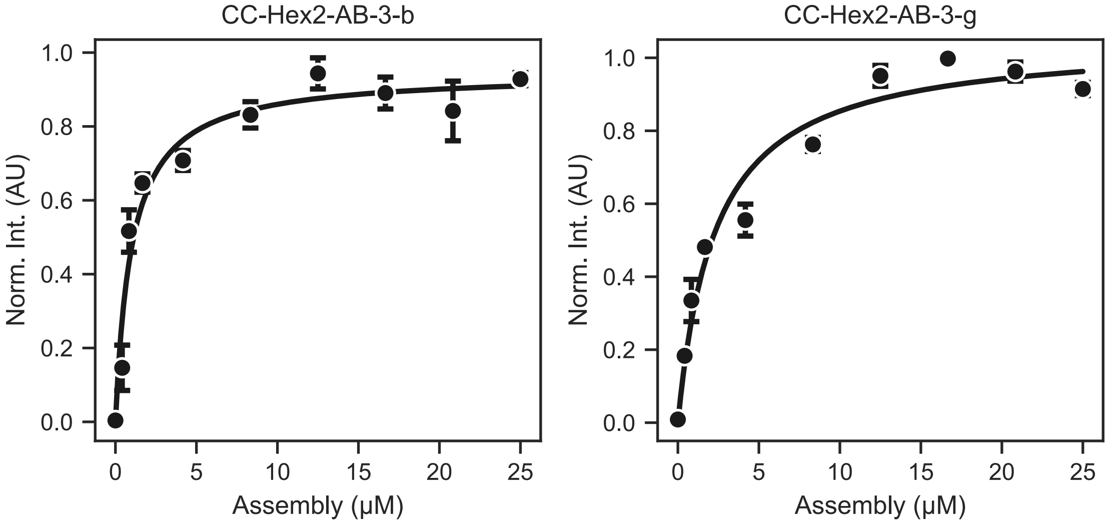

Figure S11. Saturation binding curves with DPH for tested peptides. Plots show mean fluorescence data (markers) with standard deviations (error bars). N = 3. Curves represent fits to a single site binding model. Peptide concentration is converted to αHB assembly concentration by the oligomeric state. CC-Hex2-AB-3-b results: KD = 6.0 μM (± 1.7), R2 = 0.959. CC-Hex2-AB-3-g: KD = 13.9 μM (± 8.7), R2 = 0.969. Conditions: 2.5 – 150.0 μM peptide concentrations (equimolar), HBS, 1 μM DPH, 5 % v/v DMSO.

1.5 AlphaFold3 computed structure models

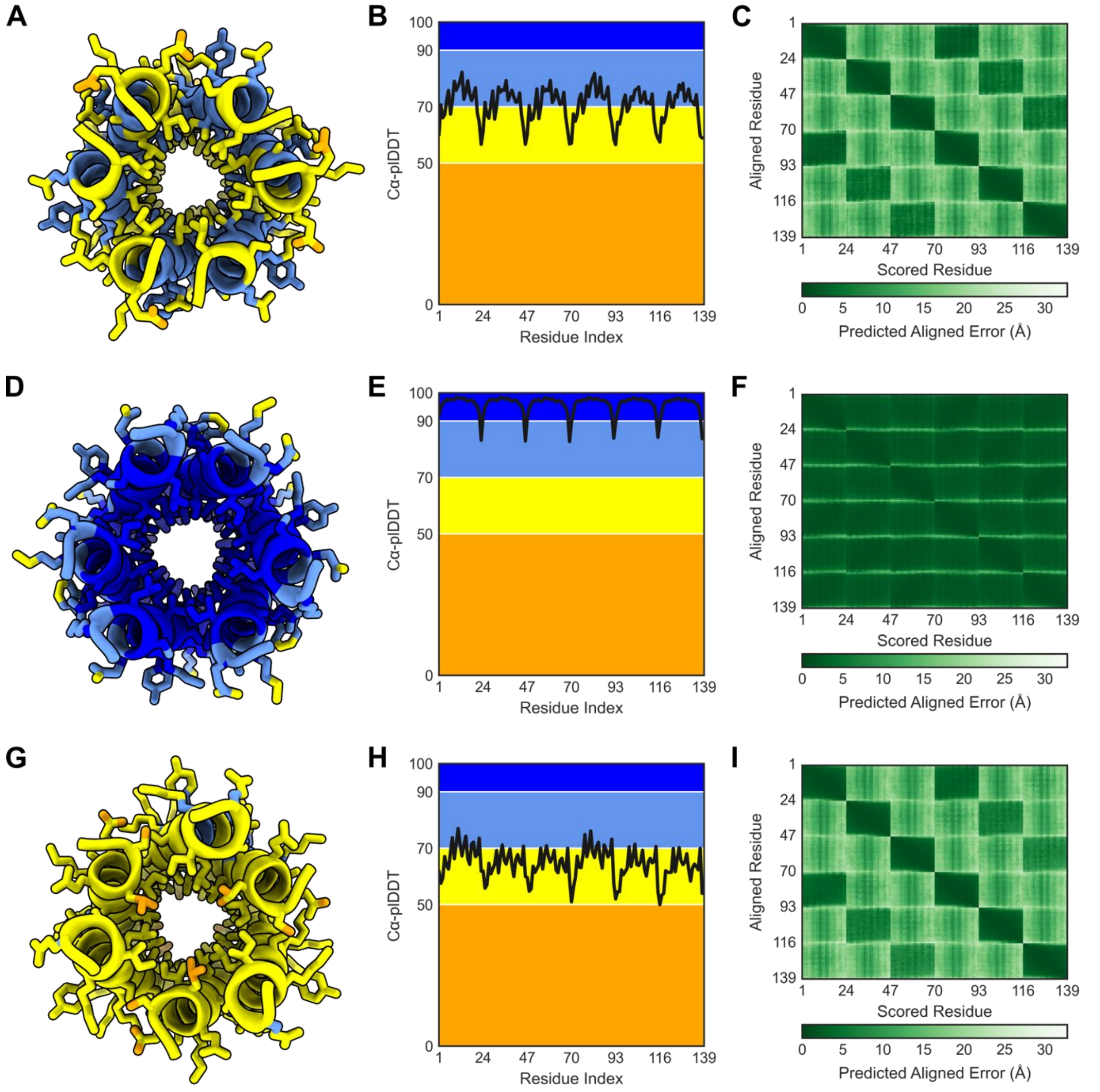

Figure S12. AlphaFold3 results for CC-Hex2-AB-3-***b*** (A – C), CC-Hex2-AB-3-***c*** (D – F) and CC-Hex2-AB3-3-***g*** (G – I). (A,D,G) All-atom prediction colored by atom plDDT confidence (very low: plDDT < 50, orange; low: 50 < plDDT < 70, yellow; confident: 70 < plDDT < 90, light blue; very high: 90 < plDDT, blue). (B,E,H) The Cα-plDDT scores for each residue. Patches reflect confidence bins as described for A,D,G. (C,F,I) The predicted aligned error (pAE) at each residue index.
